# Analysis regarding the impact of ‘fake news’ on the quality of life of the population in a region affected by earthquake activity. The case of Romania–Northern Oltenia

**DOI:** 10.3389/fpubh.2023.1244564

**Published:** 2023-12-01

**Authors:** Flavius Cristian Mărcău, Cătălin Peptan, Vlad Dumitru Băleanu, Alina Georgiana Holt, Silviu Adrian Iana, Victor Gheorman

**Affiliations:** ^1^Faculty of Education, Law and Public Administration, “Constantin Brâncuși” University of Târgu Jiu, Târgu Jiu, Romania; ^2^Faculty of Medicine, ‘Carol Davila’ University of Medicine and Pharmacy, Bucharest, Romania; ^3^Doctoral School Economics II, Bucharest University of Economic Studies, Bucharest, Romania; ^4^Faculty of Medicine, University of Medicine and Pharmacy of Craiova, Craiova, Romania

**Keywords:** “fake news”, quality of life, earthquake, authority, security

## Abstract

**Purpose:**

The study aims to examine the impact of the wave of seismic activity in the northern region of Oltenia (Gorj County, Romania) in February 2023 and the belief in ‘fake news’ (circulated regarding causality, manifestations, and future developments of the seismic activity) on the quality of life of the affected population. It was considered opportune to conduct this study, given the novelty of such a situation, as the mentioned geographical area is not known to have a high seismic risk.

**Methods:**

The study was built based on the questionnaire to which 975 respondents, present/residing in Gorj County during the earthquakes and at least 14 days after, and with a minimum age of 18 years, responded. The data was collected between February 27, 2023, and March 31, 2023, at a reasonable time interval from the recording of the first seismic event in the region, assuming that the respondents’ opinions regarding the negative impact of seismic events on societal life are well crystallized. The aim was to obtain information and analyze it in order to establish the respondents’ perception regarding the negative effects of seismic activity and the elements of “fake news” promoted in this context on the quality of life of individuals in the region.

**Results:**

Our study indicates that individuals who are not concerned, due to their disbelief in “fake news” information, about the possibility of new strong earthquakes in the mentioned area feel the best physically, having an average satisfaction level of 82.80 (with a standard deviation of 19.70) on the WHOQOL-BREF scale. On the other hand, those who believed in the fake news experienced the lowest levels of psychological well-being, with an average satisfaction of 60.80 (and a standard deviation of 21.98). The WHOQOL-BREF is an instrument that assesses the quality of life across four distinct domains, and this study emphasizes the importance of accurate and trustworthy information for people’s well-being.

**Conclusion:**

The results of the study highlight that the quality of life indicators of people in the geographic area affected by the wave of seismic movements are negatively impacted due to the release of “fake news” in the public domain regarding the cause of seismic movements in Gorj county (and the previous earthquakes in Turkey) and their future manifestations and developments (the possibility of high magnitude seismic movements), as well as the lack of information provided by the public authorities on the issue at hand (causes, effects, future manifestations, management measures).

## 1 Introduction

The effects of seismic movements, beyond the material damage they cause, also have societal implications, intensifying the uncertainty, fear, and anxiety of the population, while also disrupting everyday life (1). This unwelcome reality is further exacerbated by the spread of “fake news” in the public domain in such instances (2). The situation is also facilitated by the conveniences brought by the phenomenon of globalization (the free flow of information online). It is further amplified by the disinformation caused by the circulation of “fake news,” which can be attributed to human nature, as people seek clear answers and directions in challenging situations (like those caused by earthquakes) when the state authorities cannot provide them immediately (3).

“Fake news,” trying to provide answers to complex questions, fills the knowledge gap of the population in crisis situations, like those caused by seismic movements. People are inclined to believe information that confirms their pre-existing fears or beliefs, a phenomenon known as “cognitive confirmation” (4). In a crisis situation, this mechanism can be intensified.

### 1.1 Context

The geographical area located in northern Oltenia (Gorj County) has experienced, starting from February 13, 2023, until the present (August 15, 2023), a wave of over 3,500 seismic movements (two earthquakes with magnitudes of 5.2 and 5.7 on the Richter scale, with an interval of approximately 22 h, followed by smaller intensity aftershocks, all at shallow depths - under 20 km) (5, 6), classified in the category of natural disaster risks (7), whose frequency of occurrence and magnitude are subject to the theory of hazard.

**Figure 1:**
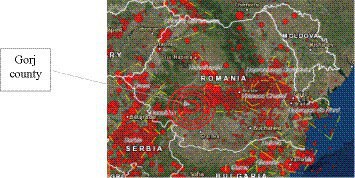
Geographical area affected by seismic movements (Northern Oltenia) (8).

We mention the fact that the mentioned geographical area (Figure 1) is not known to be characterized by high seismic risk and has not faced such a casuistic for over eight decades, even though at the national level, the seismic zone of Vrancea has been notable for its significant seismic activity, the most prominent being the earthquakes of 1940 and 1977, with a magnitude of 7.4 on the Richter scale, which caused significant material damage and loss of human lives (1).

In this context, the recent seismic movements recorded in Gorj County have negatively influenced the quality of life indicators for the affected population, both due to the induced state of panic and anxiety, as well as the material damages caused to elements of civil infrastructure (headquarters of public administration institutions, educational units, national cultural heritage sites, local entrepreneurial environment, etc.) (9–12).

In Romania, public communication in emergency situations (including those resulting from natural disasters) is institutionally regulated through the National Strategy for Public Communication and Information in Emergency Situations (13). On the other hand, public communication in such situations represents one of the priority objectives of the General Inspectorate for Emergency Situations (14), the national institution with competencies in the field of managing the discussed issues.

The lack of trust of the population of Romania in the authority of state institutions–manifested in recent years, in the context of the crises that have affected the country (12, 15)–and the deficiencies in public communication by representatives of competent state authorities (the National Institute for Earth Physics and the General Inspectorate for Emergency Situations) during the occurrence of seismic movements in the geographical area of northern Oltenia, have created favorable conditions for the dissemination in the public space, by regional and national media channels, of “fake news” type information regarding causality, manifestations, and future evolution of seismic movements. In this context, considering the impact of mass media and their high credibility among the population of Romania (16, 17), the dissemination of “fake news” information has intensified the state of uncertainty, panic, and anxiety among the population in the region, significantly affecting its quality of life.

It should also be mentioned that the effects on humans and the environment, determined by the natural disasters produced by the seismic movements that humanity has faced more and more in recent years, have made environmental security and the health of the population one of the most important dimensions of national security at the level of state entities. The framework document that regulates this issue, from the perspective of its importance for Romania’s national security for the period 2021–2024, is the National Strategy for the Defense of the Country - “*Together, for a safe and prosperous Romania in a world marked by new challenges*,” which includes references regarding the persistence of some risks of a social nature, affecting including the health status and quality of life of the population, determined by natural disasters, the degradation of environmental factors, etc. (18).

### 1.2 The analysis of the “fake news”

#### 1.2.1 General framework

“Fake news” is a term that has strongly entered public discourse in recent years, but the phenomenon itself is not new. It should be noted that the definition of this phenomenon has evolved over time (19). Today, we can say that “fake news” refers to the deliberate dissemination of false, misleading, or distorted information, with the aim to influence, manipulate, or misinform the public (3, 20). Sample et al. (4) distinguish between simple information and its use as “weapons” in communication, highlighting the shift from an information-based logic to one based on identity. Information, in the traditional sense, refers to current data about a situation or system and has a temporary, contextual, and descriptive value (21, 22). In traditional politics, accurate information ensures the knowledge necessary for decision-making at an individual or institutional level. In the modern era, information disturbances persist and intensify, contributing to the rise in societal polarization. Edson et al. (23) emphasize that fake news is opposite to “real news,” which plays an essential role in defining events and differentiating reality from falsehood. The veracity of the information dictates a certain conduct of the information sender, regulated by norms and routines established over time. Producers of “fake news,” although aiming to mimic these regulations, often evade them to capture readers’ attention, with sensationalism and ephemerality having a profound impact on the receiver of such information.

To deeply understand the nature and impact of “fake news,” it’s essential to conceptually analyze its various dimensions, considering there are different typologies or categories of misinformation, which differ based on intention, the nature of the false information, and methods of propagation:

1. *Misinformation*. This represents the accidental or unintentional spread of false information (24). People who disseminate misinformation do not do so with the deliberate intention of deceiving (25); rather, they may genuinely believe that the information is accurate (26);
2. *Disinformation*. Contrary to misinformation, disinformation involves the deliberate spread of false information with the intent to mislead or manipulate (27). This type of fake news is created and spread with the clear purpose of deceiving (28, 29);
3. *Satire and parody*. Although generally created for entertainment and not to misinform, they can be misinterpreted by certain audiences as being real news or information (30). Without the proper context, satire can be mistaken for reality;
4. *Biased or slanted news*. Such news might be based on facts but presented in a manner that favors a particular viewpoint, ideology, or agenda (31). By selectively omitting details or emphasizing some aspects over others, they can provide a deceptive perspective on reality (32)
5. *Fabrications and forgeries*. This involves creating or altering documents, images, audio, or video recordings to deceive. For instance, a doctored photo or an edited audio recording to distort the truth (33);
6. *Clickbait*. These are headlines or content primarily created to draw attention and generate clicks or views, even if the presented information is exaggerated or misleading (34);
7. *Conspiracy theories*. These narratives suggest the existence of secret actions orchestrated by powerful groups or individuals, often without solid evidence or relying on anecdotal evidence (35).

Certainly, the future will bring us new dimensions of the “fake news” phenomenon that will become increasingly difficult to detect and counteract. The dissemination in the public space of “fake news” information, aiming to create “an alternative and altered public reality by interested actors,” capable of inducing false perceptions in public opinion about a factual reality (including issues related to seismic movements) and subsequently supporting that perception (2), represents a reality faced by today’s society (36–38).

The mechanisms through which “fake news” spreads are also crucial for understanding their impact (39). Social media platforms (40), for example, are designed to amplify information that elicits strong emotional reactions (41–43). Hence, an alarmist report about an impending earthquake based on false information will likely be disseminated more quickly and broadly than a balanced and well-researched analysis of the situation. The algorithms of these platforms, in their attempt to keep users engaged, can exacerbate the problem by showing them more content similar to what they have previously seen and reacted to (44). In addition to human nature and technological mechanisms, there are cultural factors that can influence people’s susceptibility to “fake news.” In societies where trust in public institutions is low or where traditional media is viewed skeptically, fake news might seem more credible than official sources (45).

The social component cannot be overlooked either. Information often spreads in communities through personal relationships, whether directly or online. Within these social networks, there’s a tendency to trust information from trusted sources - friends, family, colleagues. If these individuals share or believe in “fake news,” it’s likely that others in their circle will accept that information as true (46).

#### 1.2.2 Description of the “fake news” phenomenon in the context of seismic movements in Northern Oltenia

The issue of seismic movements in Romania has been primarily addressed in the national specialized media after the earthquake with a magnitude of 7.4 on the Richter scale, on March 4, 1977, from the perspective of determining factors, manifestations, consequences, and measures to mitigate negative effects (1, 47–49).

Internationally, there are recent studies addressing similar issues in various geographical areas of the world (50–52), including from the perspective of disaster risk reduction and increasing resilience to the danger of seismic movements (53–55).

It is scientifically demonstrated that seismic movements are an essential factor influencing the quality of life of the population in the affected regions (56–58).

On the other hand, recent research tries to elucidate aspects related to the concerns of the scientific world in the field, about the information that is considered “fake news” (disinformation), about those on which there is no consensus regarding their nature (true, false), or those still under scientific debate, aiming to derive conclusions of interest (59).

Moreover, some studies highlight the impact of disseminating “fake news” in the context of seismic movements on the quality of life of the affected population. It is evident that these spread, especially when there is a significant information deficit that should be provided to the public by the authorities competent in disaster management, or when truncated or ambiguous information (fake news) is disseminated, triggering uncertainty, fear, and anxiety among the affected population (60, 61).

It should be noted that recent research (using the WHOQOL-BREEF tool) by authors (62) highlights the negative influences of publicizing “fake news” on people’s quality of life during crises, such as the Ukraine crisis caused by military actions undertaken on its territory by the Russian Federation after February 24, 2022.

The seismic sequence in Northern Oltenia induced panic and anxiety among the population of Gorj county right from its onset (February 13, 2023). Subsequently, due to the multitude of aftershocks, most rural property owners chose to temporarily relocate to those areas as a safety measure against potential major earthquakes. The chaos of the moment and the lack of clear information regarding the cause of the seismic events and their evolution trend allowed for the emergence of numerous misleading online details. Claims linking the seismic sequence in Northern Oltenia to similar events in Turkey [manifested by the twin earthquakes in the Gaziantep region (63) on February 6, 2023, with intensities of 7.8 and 7.5 on the Richter scale, followed by over 10,000 aftershocks (64, 65)] generated widespread panic, which facilitated the spread of false information. As a result, the online environment was flooded with information that later proved untrue, but the way they were presented increased fear among already frightened people.

This reality aligns with specialized studies which highlight concerns of individual or collective entities about releasing “fake news” on imminent seismic movements in various geographical areas, about their causes or future evolution, aimed at misleading the population, inducing a state of uncertainty, panic, and anxiety, and decreasing their trust in state authority measures for optimal crisis management (66–68).

Regarding the seismic sequence in Northern Oltenia, most of the information was released on the Facebook social platform and via the Whatsapp application, with the author/authors not being identified due to the thousands of subsequent shares. Essentially, the initial misinformation, due to the massive resharing of false messages, took on a misinformation character, with most people coming to believe in this false information due to the information void.

Among the false information proliferated online after February 13, 2023, we find the following: (1) a devastating earthquake is imminent, and the authorities are hiding the truth; (2) the authorities have information about a new earthquake of much higher intensity than the one on February 14 but withhold this information to avoid panic; (3) the earthquakes are caused by HAARP technology by the military (69–72); (4) the earthquakes are the result of shale gas exploitation in the region; (5) the earthquakes are due to coal mining from surface mines in the region, etc.

In our study, we chose to focus on the following information that proved to be false:

1. Information predicting a new high-magnitude earthquake with devastating effect. This is measured by two variables: one measuring respondents’ assessment of the likelihood of a new stronger earthquake and a variable measuring respondents’ fear of new high magnitude seismic activity (i.e., whether respondents are afraid of the occurrence of new high-magnitude seismic movements in the north of Oltenia, that could generate unwanted consequences).
2. Information regarding the causative link with recent earthquakes in Turkey;
3. information indicating belief in a high-magnitude earthquake, against the backdrop of an information deficit provided by authorities.

It should be mentioned that such “fake news” has been promoted online on other occasions, either after seismic events (73–76) or to announce future situations (77).

### 1.3 Hypothesis and objectives

Through this study, we aim to highlight the impact of the seismic movements wave in Gorj county on the quality of life parameters of the affected population, based on the following research objectives and hypotheses:

*O1*: Assessing the degree of citizens’ awareness of the seismic movements recorded in northern Oltenia.

*H1*: The population in northern Oltenia has an information/knowledge deficit about the recent seismic movements issue, which promotes their belief in “fake news” type information.

*O2*: Investigating the involvement of the authorities in public communication (by providing credible information) and in managing the issue of seismic movements, aimed at reducing the stress level of the affected population and the material damages recorded.

*H2*: The deficiencies of the authorities manifested in public communication and in managing the issue of seismic movements significantly contribute to belief in fake news.

*O3*: Examine how “fake news” type information, circulated in the context of the seismic movement wave in northern Oltenia, contributes to the decrease in quality of life indicators of the affected population.

*H3*: The population’s trust in “fake news” type information, circulated in the context of the seismic movements wave in northern Oltenia, significantly contributes to the decrease in their quality of life indicators.

## 2 Research methods

### 2.1 Participants

The study was conducted from February 27, 2023, to March 31, 2023, starting from the fourteenth day after the first seismic event recorded in Gorj County. It involved the administration of a questionnaire online through the Facebook social media platform and various websites. The questionnaire was targeted at individuals aged 18 and above who were present or had residency in the county during the pair of earthquakes and at least 14 days after these events. No data was collected on respondents’ identifiers. Participation in the research was voluntary, anonymous and unpaid, the respondents being informed about the institutional affiliation of the authors of the study and the fact that the processed data will be used for the purpose of writing a scientific study.

### 2.2 Procedure

Study participants could complete a specific questionnaire, which was built on the Google Forms platform and distributed via a dedicated web link. The questionnaire could only be completed by people who checked “Yes” to the question regarding their presence in Gorj county (during the earthquakes and at least 14 days after) and the minimum age of 18 years.

### 2.3 Measurements

The questionnaire consisted of 38 questions and was structured into two parts that aimed to: (1) Gather socio-demographic and opinion data regarding the sample respondents’ level of information, the causes and negative effects of seismic movements, and the authorities’ involvement in managing the situation; (2) Measure the quality of life of the respondents.

The information obtained from processing the questionnaire allowed for the evaluation of the respondents’ quality of life as a result of the effects of seismic movements, with the goal of validating or invalidating the research hypothesis.

#### 2.3.1 Variables measuring “fake news”

To what extent did you believe the information in the public space (released shortly after the 5.7 ML earthquake) that a much stronger earthquake would follow?; To what extent do you think that the recent wave of seismic movements in northern Oltenia is related to similar events that took place previously in Turkey?; To what extent do you believe that the information from the public sphere stating that a larger magnitude earthquake is imminent has affected your peace of mind?;

#### 2.3.2 Awareness of seismic movements

The degree of access to credible resources/materials regarding the wave of seismic movements in northern Oltenia.

Involvement of the authorities in public communication: To what extent do you think that the local authorities’ interventions were able to limit/recover the material damage?; To what extent do you think that the interventions of the local authorities were likely to contribute to reducing the stress level of the affected population (by providing credible and reassuring information)?

#### 2.3.3 Quality of life

To assess the participants’ quality of life, the WHOQOL-BREF (78) measurement tool was employed. This is a shortened version of the WHOQOL-100 instrument, both developed by the World Health Organization for assessing quality of life. WHOQOL-BREF consists of 26 questions and focuses on four main domains: (1) Physical Domain, encompassing aspects related to pain and discomfort, energy and fatigue, sleep and rest, and mobility, among others; (2) Psychological Domain, covering feelings, learning, memory, concentration, self-esteem, body image, positive or negative feelings; (3) Social Domain, referring to personal relationships, social support, and sexual life; (4) Environmental Domain, addressing aspects related to physical safety and security, home environment, financial resources, availability and quality of health and social care services, opportunities for recreation/leisure activities, and transportation. Obtaining a high score in the four domains of the WHOQOL-BREF questionnaire represents an increased quality of life. The lower the scores, the more negatively the quality of life is affected. Also, a high score on one of the variables does not lead to an increased quality of life. It is necessary to obtain high scores in all four domains for the quality of life to be considered increased.

Regarding the method for constructing the indices, each question in the WHOQOL-BREF is rated on a scale from 1 to 5. The scales are structured such that a higher score indicates a better quality of life. To obtain the score for each domain, the mean of the respective items is calculated, and this mean is then multiplied by 4 to transform the score into a scale from 4 to 20. This multiplication by 4 step allows for the comparison of WHOQOL-BREF scores with those of WHOQOL-100.

The variable “Health satisfaction” is actually one of the two questions in WHOQOL-BREF that are not attributed to a specific domain. This question evaluates an individual’s overall satisfaction with their own health. It is based on the individual’s personal perception and not on an objective measure of health status.

Data verification, cleaning activities, and calculation of scores for the major quality of life evaluation domains were performed using the WHOQOL User Manual (79).

### 2.4 Statistical data analysis

The statistical processing of the data obtained through the applied questionnaire was carried out by running the Microsoft Office Professional Plus 2021 program and IBM SPSS Statistics 26, installed on a computer equipped with the Windows 11 Professional operating system.

The data collected through the questionnaire were centralized in an Excel file, proceeding to their visualization, extraction and statistical analysis.

The variables that were the basis of the analysis concerned the opinion of the respondents regarding: (1) the degree of information provided to citizens by the authorities and the mass media regarding the seismic movements register in Gorj county; (2) the causes and effects of these seismic movements; (3) the involvement of local authorities to reduce the stress level of the affected population by providing viable information regarding the management of the newly created situation; (4) The way in which recorded seismic movements affect the quality of life of citizens, as an element characterizing human security.

The data extracted from the questionnaire were analyzed by applying descriptive statistics, in order to determine the distribution frequencies, percentages, mean scores and standard deviation. To determine the correlation between the variables extracted from the first part of the questionnaire, the Kendell test were applied. Additionally, to validate the research hypotheses, a linear regression was applied between various variables. To compare mean differences, the t-test was applied. Statistical significance was established as a *p* value of less than 0.05.

## 3 Results

### 3.1 Characteristics of the respondents

The questionnaire was applied to a number of 975 people, their main socio-demographic data being presented in Table 1.

**Table 1:** Socio-demographic data of the respondents.

Age	Sex				Environment of residence				Educational level			
	Female		Male		Urban		Rural		Middle and high school		University studies	
	*N*	%	*N*	%	*N*	%	*N*	%	*N*	%	*N*	%
18–66+	699	71.6	276	28.3	608	62.3	367	37.6	380	38.9	595	61

Table 1 indicates that there is a majority of respondents with university studies, women and people living in urban settings in the sample.

### 3.2 Respondents’ awareness of seismic motions

This section relates to the first objective of the paper, which is to assess the degree of citizens’ awareness of the seismic movements recorded in northern Oltenia.

#### 3.2.1 Access to trustworthy information

It is observed that only 52.3% of respondents state that they had access to a large or very large extent to trustworthy informational resources and materials regarding the analyzed issue (See Table 2).

**Table 2:** Respondents’ level of information about seismic movements.

Keywords	Question content	Note 1(%)	Note 2(%)	Note 3(%)	Note 4(%)	Note 5(%)
Access credible resources	The degree of access to credible resources/materials regarding the wave of seismic movements in northern Oltenia.	13.3	8.7	25.5	21.6	30.7

1, very low extent; 2, low extent; 3, neutral; 4, high extent; 5, very high extent.

The study reveals that relatively close percentages of the respondents (30–35%) appreciate the degree of objectivity of the information sources. However, 81.0% of the respondents claim that the appearance in the public space of some „fake news” information, regarding the causes and effects of the seismic movements in the north of Oltenia, induces, to a large and very large extent, a state of panic among the population (See Table 3).

**Table 3:** Respondents’ perception of the veracity of information sources.

Keywords	Question content	Note 1(%)	Note 2(%)	Note 3(%)	Note 4(%)	Note 5(%)
Objective info presentation	To what extent do you think that the information sources objectively present the causes and effects of the recent seismic movement in northern Oltenia?	17.5	16.1	35.3	17.5	13.4

1, very low extent; 2, low extent; 3, neutral; 4, high extent; 5, very high extent.

#### 3.2.2 Beliefs in “fake news”

The study highlights that a 29.6% of the respondents gave credence, to a large and very large extent, to the information released in the public space by various information sources regarding the imminence of a high-magnitude earthquake in the northern Oltenia region. It is also worth mentioning that the level of conviction of the respondents regarding the occurrence of such an earthquake is close to the previously presented assessment (See Table 4).

**Table 4:** Comparative analysis of belief in fake news.

Keywords	Question content	Note 1(%)	Note 2(%)	Note 3(%)	Note 4(%)	Note 5(%)
Belief stronger quakeinfo	To what extent did you believe the information in the public space (released shortly after the 5.7 ML earthquake) that a much stronger earthquake would follow?	28.3	16.9	25	13.9	15.7
Likelihood stronger quake	To what extent do you consider that an earthquake with a magnitude greater than 5.7 ML is likely to occur in the immediate future?	29.3	17.5	28.2	10.8	14
Relation turkey events	To what extent do you think that the recent wave of seismic movements in northern Oltenia is related to similar events that took place previously in Turkey?	22.9	15.3	31.2	16.1	14.2
Fear new high magnitude seismic activity	Are you afraid of the occurrence of new high-magnitude seismic movements in the north of Oltenia that could generate unwanted consequences?	9.7	8.8	18.6	15.9	46.8

1, very low extent; 2, low extent; 3, neutral; 4, high extent; 5, very high extent.

### 3.3 Respondents’ perception of the authorities’ action to limit the negative effects of seismic movements in the North of Oltenia

This section relates to the second objective of the study, which is to investigate the involvement of the authorities in public communication (by providing credible information) and in managing the issue of seismic movements, aimed at reducing the stress level of the affected population and the material damages recorded.

In the context of material damages caused by the seismic movements in northern Oltenia, 41.9% of respondents believe that the interventions by local authorities have only to a small and very small extent been able to limit/recover the damages. On the other hand, 52.0% of respondents consider that the interventions by local authorities have contributed to reducing the stress level of the affected population only to a small and very small extent (See Table 5).

**Table 5:** Respondents’ perception regarding the intervention of state authorities.

Keywords	Question content	Note 1(%)	Note 2(%)	Note 3(%)	Note 4(%)	Note 5(%)
Local authority damage control	To what extent do you think that the local authorities’ interventions were able to limit/recover the material damage?	22.3	19.6	29.8	16.2	11.9
Stress reduction by authorities	To what extent do you think that the interventions of the local authorities were likely to contribute to reducing the stress level of the affected population (by providing credible and reassuring information)?	29.9	22.1	25.1	12.8	9.9
Specialists explanation effectiveness	To what extent do you think that the interventions of specialists from the Institute for Earth Physics were able to explain the causes of the seismic movements recently produced in the north of Oltenia?	23.5	21.3	31.2	15.1	8.6

1, very low extent; 2, low extent; 3, neutral; 4, high extent; 5, very high extent.

The Kendall correlation test indicates a very strong connection between the high level of trust in “fake news” information and the participants’ belief in the lack of credible information provided by competent institutions (see Table 6), thus supporting Hypothesis 2.

**Table 6:** Correlation between the level of trust in “fake news” information and the lack of information from competent institutions.

Kendell	Participants who gave scores of 1 and 2 for both variables (belief stronger quakeinfo and stress reduction by authorities)***	Participants who gave scores of 4 and 5 for both variables (belief stronger quakeinfo and stress reduction by authorities)***
Corelation coefficient	0.911**	0.929**
Sig. (2-tailed)	0.000	0.001

**Correlation is significant at the 0.01 Note (2-tailed). ***1, To a very small extent/2, To a small extent/4, To a large extent/5, To a very large extent.

Regarding the causality of seismic movements in northern Oltenia, 30.3% of respondents believe, to a great and very great extent, that they are related to seismic movements previously occurring in Turkey. It is worth noting that a 44.8% of respondents believe that the interventions by specialists from the Institute of Earth Physics have only to a small and very small extent helped explain the causes of the recent seismic movements in northern Oltenia (See Table 5).

The National Institute for Earth Physics (NIEP) is a Romanian research institution that specializes in geophysics and seismology. Its primary focus is on the study of earthquakes, seismic risk assessment, and the Earth’s internal structure. The institute plays a crucial role in monitoring seismic activity in Romania and the surrounding regions, providing valuable data for both scientific research and practical applications in civil protection and urban planning. NIEP is responsible for operating the Romanian National Seismic Network, which consists of numerous seismic stations across the country. This network enables the institute to detect and analyze seismic events in real-time, contributing to a better understanding of seismic hazards in Romania.

### 3.4 Analyzes of the relationship between beliefs in fake news and quality of life

#### 3.4.1 Respondents’ quality of life

This section relates to the third objective of the study, which is to examine how “fake news” type information, circulated in the context of the seismic movement wave in northern Oltenia, contributed to the decrease in quality of life indicators of the affected population.

The perceived level of quality of life by participants, as a result of the influences of seismic movements on them, varies depending on the factors taken into consideration. Regarding the question “How do you rate your quality of life during the seismic movements in Gorj?,” the average responses, on a scale from 1 to 5, are around the value of 3.04 ± 1.166.

The average values characterizing the four major domains of highlighting participants’ quality of life (Physical, Psychological, Social, and Environment), specific to the WHOQOL-BREEF measurement tool applied in this study, are situated, on a scale from 0 to 100, in the range of 67.40 ± 20.21 and 77.26 ± 21.09 (See Table 7).

**Table 7:** Descriptive statistical analysis of the quality of life across the entire sample, according to the four major domains.

	*N*	Mean	Std. deviation
ENVIR	975	67.40	20.21
PHYS		68.64	20.10
PSYCH		77.26	21.09
SOCIAL		71.31	23.72

Within the WHOQOL-BREF assessment framework used in this study, a higher score indicates a higher perceived quality of life. Scores closer to 100 reflect a more favorable self-assessment in the respective domains of Physical, Psychological, Social, and Environmental quality of life. Conversely, scores closer to 0 indicate a lower perceived quality of life. Therefore, the average scores reported in Table 8, ranging from 67.40 to 77.26, suggest a moderately high quality of life among the participants.

**Table 8:** Respondents’ perception of the effects of information circulated in the public space.

Keywords	Question content	Note 1(%)	Note 2(%)	Note 3(%)	Note 4(%)	Note 5(%)
Info impact peace mind	To what extent do you believe that the information from the public sphere stating that a larger magnitude earthquake is imminent has affected your peace of mind?	17.1	12	20	16.9	33.9
Seismic influence life	Does the current wave of seismic movements in northern Oltenia have any influence on your life?	15.4	13	22.6	18.4	30.3
Fake news panic	To what extent do you think that fake news sources manage to panic people in Romania when they present false information about the causes and effects of the recent seismic movements in northern Oltenia?	3.1	3.3	12.6	18.9	62.1

1, very low extent; 2, low extent; 3, neutral; 4, high extent; 5, very high extent.

#### 3.4.2 Respondents’ perception of the effects of information circulated in the public space

In the context of the respondents’ very high perception regarding the promotion of “fake news” about seismic activity in northern Oltenia, 50.8% of respondents consider that their state of tranquility has been greatly and very greatly affected. This has induced an increased sense of fear regarding the possible occurrence of new seismic movements in the region (this information that has been circulating in the public space is fake news), with 62.7% of respondents. On the other hand, 52.5% of the total respondents declare that this type of influence has been felt only to a small and very small extent (See Table 8).

Applying the Kendall’s test between the variables expressing the impact on individuals’ lives and their trust in “fake news” information highlights the presence of very strong correlations, thus supporting Hypothesis 3 (See Table 9).

**Table 9:** Correlation between variables influencing the impact on individuals’ lives and their trust in “fake news” information.

Kendell	Participants who gave scores of 1 and 2 for both variables (belief stronger quakeinfo and seismic influence life)***	Participants who gave scores of 4 and 5 for both variables (belief stronger quakeinfo and seismic influence life)***
Corelation coefficient	0.822**	0.911**
Sig. (2-tailed)	0.001	0.000

**Correlation is significant at the 0.01 Note (2-tailed). ***1, To a very small extent/2, To a small extent/4, To a large extent/5, To a very large extent.

#### 3.4.3 Bivariate relationships between quality of life aspects and key variables

In Table 10, comparisons between the four main domains are presented, taking into account the socio-demographic data of the participants and the answers provided to the specific questions in the first part of the questionnaire. The codings: 1–2 means to a small extent and 4–5 means to a high extent. The significant differences between *p*-values mean differences betweensmall extent (1–2) and high extent (4–5) agreement between each mean for each question.

**Table 10:** Comparisons between the four main domains of quality of life assessment.

		Physical health	Psychological health	Social relationship	Environmental health	Quality of life (QOL)	Health satisfaction
Belief stronger quakeinfo	1–2	82.09 (18.37)′	74.36 (17.60)′	**74.45 (22.23)**′′	70.94 (18.15)′	3.36 (1.10)′	3.97 (1.03)′
	4–5	71.00 (23.82)′	60.80 (21.98)′	**67.50 (26.30)**′′	63.57 (22.44)′	2.66 (1.25)′	3.50 (1.26)′
Likelihood stronger quake	1–2	82.41 (17.90)′	74.34 (17.89)′	**74.70 (21.48)**′′	71.60 (17.78)′	3.29 (1.10)′	3.96 (1.02)′
	4–5	71.24 (24.25)′	61.39 (22.27)′	**68.03 (26.70)**′′	62.70 (23.22)′	2.83 (1.28)′	3.54 (1.29)′
Fear new high magnitude seismic activity	1–2	82.80 (19.70)′	76.18 (17.91)′	75.32 (22.55)′	72.42 (19.45)′	3.68 (1.09)′	4.14 (1.07)′
	4–5	74.24 (22.16)′	64.67 (20.72)′	68.87 (24.88)′	64.71 (21.09)′	2.80 (1.18)′	3.56 (1.17)′
Relation turkey events	1–2	80.51 (19.33)	72.03 (18.85)′	**73.21 (21.78)**′′	**69.09 (18.90)**′′	3.22 (1.13)′	**3.91 (1.04)**′′
	4–5	75.12 (22.26)	65.75 (20.83)′	**70.88 (25.71)**′′	**66.77 (21.88)**′′	2.90 (1.12)′	**3.66 (1.22)**′′
Specialists explanation effectiveness	1–2	**76.93 (22.41)**′′	67.99 (21.49)″	69.23 (24.79)′	64.05 (20.47)′	2.86 (1.22)′	3.60 (1.20)′
	4–5	**80.20 (18.55)**′′	70.78 (18.16)″	77.47 (21.37)′	74.71 (19.32)′	3.36 (1.16)′	4.07 (1.01)′
Stress reduction by authorities	1–2	75.77 (22.71)′	66.79 (21.51)′	67.74 (24.96)′	63.23 (20.72)′	2.83 (1.20)′	3.58 (1.19)′
	4–5	81.53 (17.53)′	71.76 (19.02)′	78.26 (21.62)′	75.25 (19.01)′	3.36 (1.15)′	4.06 (1.04)′

Mean(SD), *t* test used for *p* value. 1, To a very small extent; 2, To a small extent; 4, To a large extent; 5, To a very large extent; ′ - *p* < 0.05; ″ - *p* ≥ 0.05. A single apostrophe (′) is for statistical significance *P* less than 0.05 (*p* < 0.05), and two apostrophes (″) for statistical significance *p* greater than or equal to 0.05 (*p* ≥ 0.05).

### 3.5 Multivariate analyzes

#### 3.5.1 Multivariate analysis of factors influencing respondents’ beliefs in “fake news”

In this section, we Hypothesis 1 (H1): The population in northern Oltenia has an information/knowledge deficit about the recent seismic movements issue, which promotes their belief in “fake news” type information.

The multiple regression analysis presented in Table 11 aimed to evaluate the influence of demographic variables and access to informational resources on the belief that a stronger earthquake will occur. The model included “Likelihood stronger quake” as the dependent variable and examined the impact of independent variables: sex (male vs. female), type of residence (urban vs. rural), educational level (middle/high school vs. university), and access to credible information resources.

**Table 11:** Linear regression between the dependent variable Likelihood stronger quake and the independent variables sex, residence, education and access credible resources.

Model			Unstandardized coefficients		Standardized coefficients	*t*	Sig.	95.0% Confidence interval for B	
			B	Std. Error	Beta			Lower bound	Upper bound
	(Constant)		2.937	0.193		15.181	0.000	2.557	3.317
	Sex: male vs. female		0.627	0.095	0.206	6.624	0.000	0.441	0.812
	Residence: urban vs. rural		0.014	0.091	0.005	0.150	0.881	−0.164	0.192
	Education: middle/high school vs. University		−0.358	0.084	−0.132	−4.245	0.000	−0.523	−0.192
	Access credible resources		−0.053	0.031	−0.053	−1.697	0.090	−0.115	0.008
Dependent variable: likelihood stronger quake *N* = 975									
Model	*R*	R square	Adjusted R square	Std. Error of the estimate	Change statistics				
					*R* square change	F Change	df1	df2	Sig. F Change
	0.251	0.063	0.060	1.330	0.000	0.023	1	970	0.881
Predictors: (Constant), Sex, Residence, Education and Access credible resources									

Firstly, the dependent variable “Likelihood stronger quake” shows an unstandardized coefficient (B) of 2.937, indicating a significant influence on the target variable. The standard error of 0.193 is relatively small, suggesting a fairly good precision of the estimation. The high t-value (15.181) and the significance (Sig. = 0.000) undoubtedly confirm the statistical importance of this variable. The confidence interval, ranging from 2.557 to 3.317, increases confidence in the stability of the estimation.

Analyzing the independent variables, we observe how Gender (male vs. female) has a B coefficient of 0.627, suggesting that gender has a significant positive impact on the probability of anticipating a stronger earthquake. The t-value of 6.624 and the statistical significance (Sig. = 0.000) confirm the importance of this variable. The confidence interval, from 0.441 to 0.812, indicates that gender consistently influences this probability.

Residence (urban vs. rural) shows a near-zero coefficient (B = 0.014) and a very small t-value (0.150), suggesting that the type of residence (urban or rural) does not have a significant impact on the probability of anticipating a stronger earthquake. This is also confirmed by the statistical significance (Sig. = 0.881), which is well above the conventional threshold for significance.

For the variable Education (middle/high school vs. University), we have a negative coefficient (B = −0.358), indicating that higher education (university) is associated with a lower probability of anticipating a stronger earthquake compared to middle/higher education. The t-value of −4.245 and the statistical significance (Sig. = 0.000) support this conclusion. The negative confidence interval (−0.523 to −0.192) confirms the direction and stability of this influence.

Access to credible resources provides a B coefficient of −0.053, suggesting that access to credible resources has a small, although negative, impact on the probability of anticipating a stronger earthquake. However, the t-value of −1.697 and the borderline statistical significance (Sig. = 0.090) indicate a less robust influence compared to other variables.

Thus, the results suggest that factors such as gender and education level have a significant impact on the perception of earthquake risk, while residence and access to credible resources appear to have a lesser influence.

The statistical model analyzed for predicting “Likelihood stronger quake” shows limited efficiency. The R coefficient of 0.251 indicates a weak correlation between the independent variables and the dependent variable. R Square, only 0.063, suggests that the independent variables explain approximately 6.3% of the variation in “Likelihood stronger quake” leaving most of the variation unexplained by the model.

To test Hypothesis 2 (H2): The deficiencies of the authorities manifested in public communication and in managing the issue of seismic movements significantly contribute to belief in fake news, we conducted a multivariate linear regression (Table 12) to explore the impact of various independent variables on one of the variables we use to measure beliefs in fake news. The dependent variable in this model was “Fear new high magnitude seismic activity” which Is one of the variables measuring respondents’ belief in fake news. The independent variables included in the model were sex (male vs. female), residence (urban vs. rural), education level (middle/high school vs. university), and access to credible resources.

**Table 12:** Linear regression between the dependent variable stress reduction by authorities and the independent variables sex, residence, education and access credible resources.

Model			Unstandardized coefficients		Standardized coefficients	*t*	Sig.	95.0% confidence interval for B	
			B	Std. Error	Beta			Lower bound	Upper bound
	(Constant)		1.707	0.185		9.213	0.000	1.343	2.070
	Sex: male vs. female		0.368	0.085	0.127	4.334	0.000	0.201	0.535
	Residence: urban vs. rural		0.197	0.081	0.073	2.426	0.015	0.038	0.357
	Education: middle/high school vs. University		−0.409	0.078	−0.159	−5.254	0.000	−0.562	−0.257
	Access credible resources		0.321	0.028	0.334	11.395	0.000	0.266	0.377
Dependent variable: “Fear new high magnitude seismic activity” *N* = 975									
Model	*R*	R square	Adjusted R square	Std. Error of the estimate	Change statistics				
					R square change	F Change	df1	df2	Sig. F Change
	.409^a^	0.167	0.164	1.194	0.167	48.763	4	970	0.000
Predictors: (Constant), sex, residence, education and access credible resources									

The constant, which reflects the perceived stress reduction by authorities when all independent variables are at zero, is significantly different from zero (*B* = 1.707, SE = 0.185, *p* < 0.001) with a 95% confidence interval from 1.343 to 2.070. This suggests a moderate baseline belief in the authorities’ ability to reduce stress in the absence of other variables. Gender was found to be a significant predictor, with males perceiving a greater reduction in stress by authorities compared to females (*B* = 0.368, SE = 0.085, *p* < 0.001), Beta = 0.127. This indicates a gender difference in the perception of authorities’ effectiveness. Type of residence also significantly predicted perceived stress reduction, with urban residents believing more strongly in the authorities’ ability to reduce stress than rural residents (*B* = 0.197, SE = 0.081, *p* = 0.015), Beta = 0.073. Education level had a significant negative impact on the perceived effectiveness of stress reduction by authorities (*B* = −0.409, SE = 0.078, *p* < 0.001), Beta = −0.159. This suggests that individuals with university-level education are less likely to believe in the authorities’ capacity to mitigate stress compared to those with middle/high school education. Access to credible resources was a significant positive predictor (*B* = 0.321, SE = 0.028, *p* < 0.001), Beta = 0.334, indicating that individuals who have access to credible resources tend to have more belief in the authorities’ stress reduction efforts.

The multivariate linear analysis reveals that 16.7% of the variation in the dependent variable can be explained by the model’s independent variables, with an adjusted R-squared of 0.164.The standard error of 1.194 implies that the model’s predictions are relatively precise. The R-squared change appears to be a misreported value since it should not be negative. The significant F Change indicates that the independent variables significantly contribute to the model, showing that they are useful in predicting the dependent variable.

#### 3.5.2 Multivariate analysis of factors influencing respondents’ quality of life

In Table 13, for testing Hypothesis 3 (H3), linear regression between the dependent variable Quality of Life (QOL) and the independent variables “belief in the information indicating the imminent occurrence of an earthquake much larger than the 5.7 magnitude one,” “confidence that a much larger earthquake is impending,” “disturbance of tranquility due to information forecasting a new earthquake,” “fear of the occurrence of a new earthquake,” and “Events in Oltenia are connected with recent events in Turkey” is presented.

**Table 13:** Linear regression between the dependent variable QoL and the independent variables belief stronger quakeinfo, likelihood stronger quake, info impact peace mind, fear new high magnitude seismic activity and relation turkey events.

Model			Unstandardized coefficients		Standardized coefficients	*t*	Sig.	95.0% confidence interval for B	
			*B*	Std. Error	Beta			Lower bound	Upper bound
	(Constant)		4.144	0.290		14.266	0.000	3.572	4.715
	Sex: male vs. female		−0.364	0.107	−0.136	−3.401	0.001	−0.574	−0.154
	Residence: urban vs. rural		0.204	0.093	0.083	2.179	0.030	0.020	0.387
	Education: middle/high school vs. University		−0.076	0.094	−0.032	−0.800	0.424	−0.261	0.110
	(4) Info impact peace mind		−0.111	0.037	−0.134	−3.039	0.002	−0.183	−0.039
	(5) Fear new high magnitude seismic activity		−0.202	0.039	−0.231	−5.166	0.000	−0.279	−0.125
	(6) Likelihood stronger quake		−0.018	0.046	−0.021	−0.392	0.696	−0.108	0.072
	(7) Relation turkey events		−0.023	0.034	−0.026	−0.687	0.492	−0.090	0.043
Dependent variable: QOL *N* = 975									
Model	*R*	R square	Adjusted R square	Std. error of the estimate	Change statistics				
					*R* square change	F change	df1	df2	Sig. F change
	0.398^d^	0.159	0.153	1.087	−0.001	0.640	1	589	0.424
Predictors: (Constant), belief stronger quakeinfo, likelihood stronger quake, info impact peace mind, fear new high magnitude seismic activity and relation turkey events									

The previous table (Table 13) shows a link between the impact on respondents’ quality of life and their trust in “fake news” type information. Thus, the more individuals believe in false information, the more the quality of life indicators are negatively affected. Furthermore, the analysis elucidates that certain demographic and perceptual factors have a significant bearing on the quality of life. For instance, the fear of new seismic activity has a pronounced effect, suggesting that psychological distress linked to such fears tangibly diminishes life satisfaction. Moreover, the perceived likelihood of stronger quakes ahead correlates with a heightened sense of vulnerability, which can undermine the overall sense of well-being. Interestingly, the data indicates that urban versus rural residence also plays a role, with urban dwellers possibly facing more stressors that can impact their quality of life. Educational attainment emerges as another pivotal factor, wherein higher levels of education are associated with a better ability to discern between credible information and ‘fake news,’ thereby buffering against the negative implications of misinformation on one’s lifestyle and mental peace. Collectively, these variables highlight the complex interplay between information consumption, personal beliefs, and the socio-demographic context in shaping the quality of life amidst seismic threats.

The Adjusted R-squared value, which stands at 0.153 in our model, indicates that approximately 15.3% of the variability in the dependent variable can be accounted for by the combination of independent variables used in the analysis. This statistic is particularly useful for assessing the overall fit of the model, as it accounts for the number of variables included and thus avoids the potential for overestimating the model’s explanatory power. A higher Adjusted R-squared value would suggest a better fit of the model to the data, whereas a lower value indicates that there are other, unaccounted-for factors that may be influencing the dependent variable or that the included predictors are not sufficiently capturing the relationships within the data.

We also considered combining the variables measuring fake news into a sumscore index. The Cronbach’s Alpha result obtained for the combined index of the three variables is 0.334. This value indicates a low cohesion among the items of the index, suggesting that the included variables are not sufficiently homogeneous in measuring the same concept. A Cronbach’s Alpha of 0.334 emphasizes that the variables combined in this index do not strongly correlate with each other. This implies that each variable measures different aspects.

## 4 Discussions

The study reveals that the quality of life of respondents has been significantly affected, both due to the heightened seismic activity in Gorj County and the dissemination of “fake news” information in the public domain regarding the causality, manifestation, and potential evolution of seismic movements in the region. Since the respective geographic area has not faced such an issue recently, the pair of earthquakes on February 13 and 14, 2023, and the aftershocks that continue until the present (August 15, 2023) have generated a high psychological pressure among the affected population. This aligns with the conclusions of specialized studies addressing similar issues in other geographic areas (80). Additionally, in our study, concerning the construct of quality of life, we find low indices in the “environmental” and “psychological” domains. These aspects were possible due to the earthquakes and the respondents’ concerns about potential damage to living spaces, and due to the psychological pressure and anxiety created by trusting “fake news” information.

### 4.1 Information, “fake news, “and the perception of reality (O1–H1)

Regarding the respondents’ level of information, we observe that over half (52.3%) indicate they had access to trustworthy informational resources and materials concerning the seismic movements (Table 2). Given that earthquakes pose a genuine threat to the population’s safety in the region, this statistic should serve as a foundation for future public communication initiatives by authorities during emergencies and to combat misinformation, as specialized studies also highlight (62, 81, 82). The fact that nearly 22% of respondents either did not access or did not find credible information (Table 2) is alarming and underscores an urgent need to enhance communication channels. The study results show that a staggering 81.0% of respondents believe that the promotion of “fake news” type information in the public space–referring to the causes and effects of the seismic movements in northern Oltenia–can induce panic among the population (Table 3).

In the era of information, where the spread of “fake news” has a profound impact on the public’s perception and behavior, the study reveals that 29.6% of respondents believed in the information suggesting an impending high magnitude earthquake in the region (Table 4). This belief amplifies insecurity and fear among the population, making them more susceptible to misinformation. Such circumstances can exacerbate the public’s dwindling trust in state institutions, a trend observed globally in recent years (83). This lack of trust can even jeopardize national security in times of crisis, as witnessed in Romania during the effects of the COVID-19 pandemic (84, 85), the military crisis in Ukraine (62), and the wave of seismic movements in northern Oltenia (12).

Discussions regarding Hypothesis 1 (H1) indicate a partial acceptance. It is confirmed that the population in northern Oltenia exhibits a significant baseline level of belief in information related to strong earthquakes. The analysis shows that gender has a notable statistical impact, with men being more inclined to believe in the possibility of strong earthquakes compared to women. On the other hand, place of residence and education level do not significantly contribute to this belief, indicating that demographic influences on the perception of seismic risk are limited (See Table 11).

Access to credible information does not appear to alter the belief in strong earthquakes, suggesting that other channels of information might be more relevant. The current model, having limited predictive capacity, suggests that there are unidentified factors influencing the belief in earthquake information. Consequently, a deeper exploration of the factors that shape beliefs about seismic risks is warranted, in order to enhance understanding of the phenomenon within the studied regional context.

The results obtained validate the research hypothesis (H1) concerning the information/knowledge deficit observed in the population of the geographic area affected by the recent seismic movements. This deficit tends to heighten their trust in “fake news” type information.

### 4.2 The involvement of authorities in public communication and in managing the issues of seismic movements; effects on the population’s beliefs in fake news (O2-H2)

The study results indicate that in a context where 48.7% of respondents feel the seismic movements directly affected them (Table 8), and competent institutions failed to provide relevant information about the causality of the seismic sequences (Table 8), 44.8% of respondents express increased fear about a potential causal connection with the seismic events that took place in Turkey on February 6, 2023 (Table 4). This leads to a perception among respondents of possible negative consequences, comparable to those observed in Turkey. The results imply that individuals drawing causal connections between seismic events in different parts of the world witness a decline in their quality of life. This decline can be seen as a manifestation of a general state of anxiety and unease which, even if rooted in misconceived causality and trust in “fake news” disseminated in public spaces (66, 86), affects their perception of safety and well-being.

Conversely, 44.8% of respondents highlight shortcomings in public communication by the Institute for Earth Physics (viewed as an authority in the domain) regarding the causality, impacts, and prospects of future seismic movements (Table 5). This has contributed to psychological strain, evident in the negative impact on their quality of life indicators.

The fact that 41.9% of respondents are unsatisfied with local authorities’ interventions following the damages (Table 5) indicates a significant trust deficit, which can amplify the impact of “fake news” and can erode confidence in preventive or disaster response measures, in line with research in the field (60). This is also highlighted by the high percentage of respondents (52%) who believe that local authorities’ interventions contributed to reducing the stress levels of the affected population only to a small and very small extent (Table 5).

The results underline the importance of efficient communication and actions from local authorities. The negative perception of how they handled the situation reflects on the affected population’s quality of life. This imposes the need for a proactive, transparent, and evidence-based response from authorities in crisis situations to maintain public trust and support overall well-being and counteract the effects of “fake news” circulated in context. Moreover, the Kendell correlation test indicates a very strong link between the high level of trust in “fake news” and the participants’ belief about the lack of credible information provided by the competent local institutions (See Table 6).

The examination of Hypothesis 2 substantiates the proposition that the way authorities communicate and manage information about seismic activity is crucial in shaping public belief and stress levels. The study reveals a foundational trust in the authorities’ ability to mitigate seismic stress, which varies by gender and location, with males and urban dwellers showing more confidence. Notably, those with higher education are more skeptical of the authorities’ efforts, while individuals with access to reliable information tend to trust these efforts more.

Statistical analysis indicates a significant 16.7% of ‘Quality of Life’ variance is accounted for by the model’s variables, with a strong adjusted R-squared value. Although the R-squared change is atypical, the significance of the F Change confirms the relevance of the variables chosen. This endorses the model’s effectiveness in predicting ‘Quality of Life’ in relation to seismic events and validates the hypothesis that effective authority communication is influential in public perception and stress concerning seismic risks (See Table 12).

The obtained results support the research hypothesis (H2) about the negative influences of authorities’ deficiencies in public communication and managing the issues of seismic movements on the affected population’s quality of life.

### 4.3 Confidence in false information and the impact on quality of life (O3-H3)

The study reveals that 50.8% of respondents believe that their peace of mind was significantly affected by the spread of “fake news” in the public space, which induced a heightened state of fear in 62.7% of them (See Table 8). By applying the Kendell test between variables expressing the impact on people’s lives and their trust in “fake news,” very strong correlations were found, thus supporting the research hypothesis (See Table 6).

Regarding the indicators for evaluating the quality of life, the study results (Table 7) show that the highest average satisfaction among the four WHOQOL-BREF domains is represented by the “Psychological” domain of the category of people who have the least concern about the effects of seismic movements on their quality of life (77.26 ± 21.09). Conversely, the lowest average is represented by the “Environment” domain (67.40 ± 20.21).

Scores for the four major domains, calculated based on answers to specific questions (Table 10) from the first part of the questionnaire, support the proposed research hypothesis. Specifically, individuals who believed in “fake news,” suggesting an impending earthquake greater than 5.7 on the Richter scale, had significantly lower averages across all four major domains compared to those who did not believe such information. The highest score is found in the “Physical” domain (82.09 ± 18.37) for people who did not believe false information, and the lowest in the “Psychological” domain (60.80 ± 21.98) for those who embraced such beliefs (See Table 10).

The variable assessing respondents’ perception of the likelihood of a stronger quake served as a control for the variable that measures belief in public information about an imminent, much stronger earthquake. Average scores for both questions were roughly equivalent for scores (1–2) and (4–5). In the “Environment” domain, the maximum difference for those who answered 4 and 5 was 0.87, while for those who answered 1 and 2, it was 0.66. In the “Psychological” domain, the most substantial difference, 13.56, was observed for the variable measuring belief in public information about an impending stronger earthquake. In contrast, the smallest difference, 6.67, was noted in the “Social” domain for the variable assessing the likelihood of a stronger quake (Table 10).

Those respondents who expressed concerns about potential high-magnitude seismic movements in northern Oltenia, which might have adverse outcomes, generally displayed a notably lower average across all major domains in comparison to their less concerned counterparts. Within these results, the highest value is evident in the “Physical” domain (82.80 ± 19.70) among individuals not expressing these fears (scores 1–2). In contrast, the lowest value can be found in the “Psychological” domain (64.67 ± 20.72) for those who did articulate their apprehensions (scores 4–5) about a significant seismic event in the region.

Intimately linked to this is another variable: respondents’ perceptions regarding a potential connection between the seismic activities in northern Oltenia and similar prior events in Turkey. The averages for this perception align closely with the previously discussed fear variable, with similar scores for ranges (1–2) and (4–5). Notably, the largest gap of 11.51 within the same major domain emerges in the “Psychological” sector when discussing the fear of new seismic activities in northern Oltenia. Conversely, the narrowest gap of 2.32 appears in the “Environment” domain for the variable probing into the perceived connection between seismic events in northern Oltenia and Turkey (see Table 10).

Individuals who viewed the interventions of specialists from the Institute for Earth Physics negatively (scores 1–2) in explaining the causes of the seismic movements in northern Oltenia demonstrated a significantly lower average across all major domains compared to those who viewed them positively (scores 4–5). The highest value in this regard is seen in the “Physical” domain (80.20 ± 18.55) for the positive respondents, while the lowest appears in the “Environment” domain (64.05 ± 20.47) for the negative ones (Refer to Table 10).

Closely related to the above variable is another that gages respondents’ belief in the efficacy of local authorities in mitigating the stress of the affected population due to seismic events. The average values for these two variables align closely, especially for score ranges (1–2) and (4–5). The most pronounced difference of 12.02 between averages in the same domain is evident in the “Environment” sector for the variable related to local authorities’ stress-reducing interventions. In contrast, the narrowest gap of 2.79 is observed in the “Psychological” domain concerning the specialists’ explanatory interventions (See Table 10).

In the discussions regarding Hypothesis 3, it becomes evident that individuals’ beliefs in misinformation, particularly regarding imminent larger earthquakes, significantly detract from their quality of life. The observed relationship suggests that the more one subscribes to false information about seismic risks, the greater the adverse impact on well-being. This extends beyond mere belief, affecting those who experience anxiety and fear of future earthquakes, which directly diminishes their life satisfaction. Demographic factors such as urban residency and educational attainment are also instrumental. Urban residents may encounter more stressors, affecting their quality of life more than rural residents. Higher education correlates with an increased ability to discern reliable information from fake news, offering some protection against the stress induced by misinformation.

The statistical analysis, represented by an Adjusted R-squared of 0.153, supports that these independent variables collectively account for 15.3% of the variance in quality of life. This figure confirms a moderate explanatory power of the model but also suggests the presence of other influential factors not included in the analysis. The discussion confirms Hypothesis 3 to a significant extent, indicating the importance of accurate information and the management of misinformation to safeguard public well-being, especially in the context of seismic risk perception. The findings underscore the need for effective communication strategies and education to enhance the community’s resilience against misinformation and its psychological impacts (See Table 13).

As observed, each of the variables - fear of new seismic activity, likelihood of a stronger earthquake and the relation to events in Turkey - shows a significant contribution when analyzed separately. This finding suggests that, despite potential multicollinearity among these variables, each has a distinct and significant influence on the model when considered independently.

Additionally, a clear correlation is evident between the Quality of Life indices and individuals from rural areas in relation to the variable evaluating the impact of information from the public sphere on respondents’ peace of mind. For the variable that assesses respondents’ perception of the connection between seismic movements in northern Oltenia and similar events in Turkey, a strong correlation emerges with individuals possessing a medium level of education.

Thus, the respondents’ quality of life was seriously affected due to their trust in false information predicting a new devastating earthquake with an intensity of over 5.7.

The results support the research hypothesis (H3) that respondents’ trust in “fake news” circulated in the context of the seismic movements in Gorj county significantly contributes to the decline in their quality of life indices.

## 5 Contributions to the development of public policies in the field

Since this study brings some contributions to the investigation of the quality of life of individuals affected by seismic events, as well as to establishing a causal relationship between the behavior/quality of life of citizens affected by such events and the spread of ‘fake news’ information, we believe that it could improve the debates related to the development of public policies for public health programs, especially those focused on crisis response and disaster management.

The results of the study regarding the correlation between the level of population awareness (in problematic situations such as the one analyzed) and their quality of life can contribute to supporting institutional activities at the national level to reduce disaster risks, considering the efforts to implement a specific strategy (87) aimed at ensuring community resilience through cooperation among authorities, institutions, and civil society.

On the other hand, the study results that highlight the need for authorities’ involvement in informing/advising the population in exceptional situations can also contribute to institutional efforts to implement the National Recovery and Resilience Plan in Romania, specifically the “Good Governance” section, focusing on public sector reform and strengthening the capacity of social partners. This aims to improve the decision-making system in a “predictable, evidence-based, and participatory” manner and ensure the provision of public services to the population at high-quality standards (88).

Moreover, the study results concerning the negative influences of “fake news” on the population’s quality of life can support public policies in the field of population health by combating this type of information through a comprehensive and collaborative approach, especially in crisis situations. This can be achieved through educational measures and awareness campaigns aimed at fostering citizens’ understanding of the mass media phenomenon and acquiring critical thinking skills. By doing so, it contributes to improving the resilience and quality of life of citizens.

Furthermore, the study results can make significant contributions to the national implementation efforts of the 2030 Agenda for Sustainable Development (89), particularly to Objective 3 – “Good Health and Well-being,” which aims to ensure healthy lives and promote well-being for all citizens. Additionally, it can also support Objective 16 – “Peace, Justice, and Strong Institutions,” which focuses on implementing effective, accountable, and inclusive institutions at all levels of governance.

Last but not least, considering that the health status and quality of life of the population, negatively influenced by natural disasters, represent a national security issue for Romania during the period 2021–2024 (as stated in the National Defense Strategy – “Together, for a Secure and Prosperous Romania in a World Marked by New Challenges”), the study results can provide a basis for the reconceptualization and redefinition of subsequent public policies related to national security in the mentioned field.

## 6 Limitations of the research

Our research presents certain strengths, considering it is among the first studies in Romania to address the topic of seismic movements within a defined geographic area (Northern Oltenia). However, it also has certain limitations as follows:

Firstly, the research methodology is entirely based on the analysis of data collected through an online administered questionnaire. This approach has significant implications for the representativeness and generalizability of the results (90).

One of the most evident limitations is related to the data collection environment. Not all citizens have equal access to the internet or are familiar with technology usage. This means that certain segments of the population, especially the most vulnerable or less technologically inclined, might be completely omitted from our sample (91).

Furthermore, concerns arise about the type of individuals who are more likely to respond to online questionnaires. There can be fundamental differences between those who decide to participate and those who choose to ignore the request, introducing bias into the results. For example, respondents who are more passionate or have strong opinions about our research topic might be more inclined to complete the questionnaire. This could lead to an “echo chamber” phenomenon where the questionnaire might be distributed and completed predominantly by groups that already have aligned or similar opinions (90).

Another relevant aspect is researchers’ inability to directly monitor and guide respondents’ experience in the online environment. In a traditional setting, researchers can clarify ambiguous questions or observe respondents’ behaviors and reactions, providing valuable insights. In the online version, we lack this opportunity, which means there’s an increased risk of misinterpretations or responses based on misunderstandings.

On the other hand, while online anonymity might encourage honesty in some cases, it can also give rise to less serious or even false responses, thus affecting the quality and authenticity of the collected data.

## 7 Conclusion

The results of the study highlight that the quality of life indicators for individuals in the northern region of Oltenia (Romania), affected by the intense wave of seismic activities starting from February 13, 2023, are negatively influenced due to a combination of factors, such as: the dissemination of “fake news” information in the public space regarding the occurrence of high-magnitude seismic movements; the causal links drawn between the seismic activities in Gorj County and those previously observed in Turkey; the lack of information provided by public authorities regarding the causality, effects, and future outlook of new seismic movements.

The seismic events that began in February 2023 have created significant psychological pressure on the population, given their unfamiliarity with such events recently.

The need to access credible information sources is evident, as 22% of respondents believe they did not have access to such sources during the seismic wave. This highlights the importance of transparent and factual communication from authorities and experts. This need is also determined by the fact that approximately half of the respondents are skeptical or dissatisfied with the explanations and interventions of the authorities, emphasizing the importance of more effective communication and action in crisis management.

On the other hand, there is a notable segment of respondents (81.0%) who comment on the harmful effects of “fake news” circulated in the mentioned context, believing that this exacerbates their feelings of insecurity and lack of trust in state institutions. This dynamic can have negative implications for national security in crisis situations. Additionally, the study highlights a clear correlation between the credibility given to false information and a decreased quality of their life, especially in the psychological domain.

The study results can contribute to improving the debates related to the development of public policies for public health programs, particularly those focused on crisis response, disaster management, and the impact of “fake news” on human security.

## Data availability statement

The datasets presented in this study can be found in online repositories. The names of the repository/repositories and accession number(s) can be found in the article/supplementary material.

## Author contributions

FM and CP contributed to conception and design of the study and wrote the first draft of the manuscript. FM organized the database and performed the statistical analysis. VB, AH, SI, and VG wrote sections of the manuscript. All authors contributed to manuscript revision, read, and approved the submitted version.
